# Effectiveness of Intrapartum Perineal Massage in Preventing Perineal Trauma in Nulliparous Women During the Second Stage of Labour: A Randomised Controlled Trial

**DOI:** 10.1155/ogi/1866988

**Published:** 2025-02-28

**Authors:** Obinna K. Nnabuchi, George U. Eleje, Joseph I. Adinma, Emmanuel O. Ugwu, Ahizechukwu C. Eke, Joseph I. Ikechebelu, Okechukwu C. Ikpeze, Betrand O. Nwosu, Gerald O. Udigwe, Joseph O. Ugboaja, Osita S. Umeononihu, Chukwudi A. Ogabido, Ikechukwu I. Mbachu, Chukwuemeka O. Ezeama, Richard O. Egeonu, Arinze C. Ikeotuonye, Tobechi K. Njoku, Chukwuemeka C. Okoro, Charlotte B. Oguejiofor, Ifeanyichukwu J. Ofor, Ifeoma M. Nnabuchi, Chidinma C. Okafor, Chinedu L. Olisa, Chigozie G. Okafor

**Affiliations:** ^1^Department of Obstetrics and Gynaecology, Nnamdi Azikiwe University Teaching Hospital, Nnewi, Anambra State, Nigeria; ^2^Department of Obstetrics and Gynaecology, Nnamdi Azikiwe University, Awka, Anambra State, Nigeria; ^3^Department of Obstetrics and Gynaecology, University of Nigeria Teaching Hospital, Ituku-Ozalla, Enugu, Enugu State, Nigeria; ^4^Division of Maternal and Fetal Medicine, Department of Gynaecology and Obstetrics, Johns Hopkins University School of Medicine, Baltimore, Maryland, USA; ^5^Department of Obstetrics and Gynaecology, ESUT College of Medicine, Parklane, Enugu, Enugu State, Nigeria; ^6^Department of Chemical Pathology, Nnamdi Azikiwe University Teaching Hospital, Nnewi, Anambra State, Nigeria; ^7^Department of Psychiatry, Leicestershire Partnership NHS Trust, Leicester, UK

**Keywords:** laceration, nulliparity, perineal massage, perineal pain, second stage of labour, vaginal delivery

## Abstract

**Objectives:** To determine the effectiveness of perineal massage during the second stage of labour in preventing perineal trauma in nulliparous women.

**Methods:** A randomized control trial involving 104 nulliparous women undergoing vaginal delivery. The participants were randomised into two arms (intervention-group ‘A' and control-group ‘B') in a 1:1 ratio. The intervention group had perineal massage and routine hands-on technique according to the hospital protocol while the control group received only hands-on technique. The primary outcome measure was the incidence of perineal tears during vaginal delivery, while the secondary outcome measures were the incidence of episiotomy during vaginal delivery, the mean duration of second stage of labour, and perineal pain score.

**Results:** The baseline socio-demographic and maternal characteristics of the participants were similar in both arms. The incidence of perineal lacerations in the intervention group (massage group) was significantly lower than in the control group (27 (54%) vs. 40 (81.6%); *p*=0.003). Although the incidence of episiotomy (26% vs. 44.9%; RR = 0.66; 95% CI = 0.50–0.88; *p*=0.060) was not significantly different, mean perineal pain score at 4 h postpartum (4.3 ± 0.3 vs. 6.1 ± 0.50; *p*=0.03), mean perineal pain score at 24 h postpartum (2.2 ± 0.1 vs. 4.2 ± 0.3; *p*=0.02), and mean duration of second stage of labour (83.1 ± 17.5 min vs. 94.2 ± 18.9 min; *p*=0.002) were significantly lower in the intervention-group. There was no significant difference in the neonatal outcomes (head circumference, birth weight, and Apgar scores: *p* > 0.05) between the two groups.

**Conclusion:** Intrapartum perineal massage significantly decreases the risk of overall perineal trauma, perineal pain, and duration of the second stage of labour among nulliparous parturients during the second stage of labour. Nulliparous women should be counselled on the potential benefits of intrapartum perineal massage, and obstetricians are encouraged to provide the technique to consenting women.

**Trial Registration:** Pan African Clinical Trial Registry (PACTR): PACTR 202207835155214

## 1. Introduction

The process of vaginal delivery is marked by many physical and psychological stressors [1]. These stressors are present from the beginning of the first stage of labour and reach their maximum at the second stage, which is considered the climax of the vaginal delivery process. Perineal pain from straining and tearing of the perineum and pelvic floor muscles and severe abdomen and back pain from spontaneous uterine contractions are the two most well-known causes of maternal distress during labour [1, 2].

The incidence of perineal trauma after vaginal birth varies considerably, with between 53% and 79% of women experiencing some type of perineal trauma [3, 4]. Most of these are first- and second-degree tears; the incidence of third- and fourth-degree perineal tears in women who give birth vaginally varies from 0.1% to 10.9%, depending on the study [5]. The risk factors for perineal trauma include foetal macrosomia, nulliparity, precipitous labour, malpresentation, malposition, shoulder dystocia, assisted vaginal deliveries such as forceps delivery, and vacuum extraction [6]. Even though episiotomy reduces the risk of spontaneous perineal tears, like most surgical procedures, it has some significant complications [7].

Significant short- and long-term maternal morbidity, increased hospital length of stay, and impaired mother-child bonding have all been linked to perineal trauma. The potential complications from perineal trauma include haemorrhage, haematoma, infection, vesicovaginal fistula, painful intercourse, and urine, flatus, and faecal incontinence [8, 9]. Perineal pain is frequent in nulliparous women, and it is linked to issues like insomnia and anxiety [6, 8]. These complications, no doubt, will affect a woman's quality of life and the care of the baby. Nulliparous women are more vulnerable to spontaneous perineal lacerations because of their not previously stretched perineum, and they are likely to respond positively to perineal massage, intended to decrease perineal muscular resistance [9]. Perineal massage increases elasticity of the perineum and leads to easier stretching of the muscles and less pain during childbirth [9]. Therefore, perineal massage, which is a noninvasive and affordable intervention, is worth giving a try as a preventive measure against perineal trauma [10].

Available literature also shows varying results on the effectiveness of perineal massage in preventing perineal injuries [1, 6, 9, 11–15]. While some trials during the second stage of labour have been found to reduce the incidence of perineal injuries during vaginal birth [6, 15], others have shown no evidence of a reduction in perineal injuries after the perineal massage [1, 11, 12]. According to a recent systematic study and meta-analysis by Marcos-Rodríguez, Leirós-Rodríguez and, Hernandez-Lucas perineal massage appears to be useful in preventing episiotomies and shortening the duration of the second stage of labor. However, it does not seem to be useful in reducing the frequency and intensity of perineal tears [16]. Lucena da Silva et al. and Aquino et al., conducted a similar systematic study and came to the conclusion that pregnancy-related practices, particularly perineal massage, are linked to a decreased incidence of perineal laceration [17, 18]. There is therefore a need for further well-designed randomised clinical trials in this regard to evaluate the effectiveness of perineal massage in preventing perineal trauma in nulliparous women during the second stage of labour.

We hypothesised that perineal massage would significantly prevent perineal trauma in nulliparous women during the second stage of labour compared with a standalone conventional hands-on technique. This study is aimed at determining the effectiveness of perineal massage during the second stage of labour in preventing perineal trauma in nulliparous women.

## 2. Methods

### 2.1. Study Design

The study is an unblinded, randomised, controlled trial.

### 2.2. Study Population

The study is a single-centre study conducted among eligible nulliparous participants admitted into the labour/delivery ward of Nnamdi Azikiwe University Teaching Hospital (NAUTH), Nnewi, Nigeria, following informed written consent.

### 2.3. Study Settings

The study was conducted at NAUTH, Nnewi, south-east Nigeria, from August 1, 2022, to January 31, 2023.

### 2.4. Sample Size Estimation

A power calculation was performed to determine the sample size based on data from a previous similar study investigating the proportion of perineal trauma in the massage group and control group. Thus, sample size (N) for each group was calculated using the two-tail parallel hypothesis of equivalence formula for the comparison of two proportions in a randomised clinical study. Considering a 95% confidence interval (*α* = 0.05) and a power of 80% (*β* = 0.2), the formula was used, where *p* (pooled prevalence) = proportion of perineal trauma in the intervention/massage group of a previous study by Oglak and Obut [19], proportion of perineal trauma in the control group of a previous study done by Oglak et al. = standard normal deviation for *α*, generated from the Z-scores table at a type 1 error of 5 = 1.96, and standard normal deviation for *β*, generated from the Z-scores table at 80% power = 0.84. With an attrition rate of 20%, the minimum sample size for each arm was 52 (a total of 104 participants).

### 2.5. Inclusion Criteria

There were nulliparous women, aged 18–45, singleton pregnancies, cephalic presentations, who presented in spontaneous labor at term. After inclusion, they were checked during birth again for the following additional inclusion criteria, which included 37–42 weeks of gestation (term), cephalic presentation, and in the second stage of labour (cervical os dilatation of 10 cm).

### 2.6. Exclusion Criteria

Those excluded from the study were women with female genital mutilation and multiple pregnancies. After exclusion, they were checked during birth again, and the following participants were further excluded: women with poor progress of labour, persistent fetal tachycardia, prematurity, erythematous rashes, oedema of the perineum, fetal malpresentation, participants with small for gestational age and large for gestational age. Specifically, we have also excluded women with a history of significant perineal trauma or prior caesarean section, as these could influence the outcomes of the current study.

### 2.7. Randomization Technique and Group Allocation Sequence

Randomization and allocation concealment were applied. Due to the nature of the intervention, blinding of the participants or researchers was impossible. To avoid selection bias, a computer-based random sequence generator (https://www.randomization.com) created by a statistician (who was not part of the study team) was used to randomise the participants in a 1:1 ratio using randomization blocks of 4. Sealed, nontransparent brown envelopes were marked serially from 1 to 104; each numbered envelope contained a white piece of paper labelled either ‘A' for the intervention group or ‘B' for the control group. The envelopes were handed over to a registrar (a resident doctor) who was completely unaware of the objectives of the study. The doctor enrolled and subsequently allocated participants to their groups. As the participants were being recruited, serial numbers 1 to 104 were assigned to them accordingly. The intervention group received perineal massage for a maximum duration of 10 min during the second stage of labour, while the control group did not receive perineal massage during the second stage of labour. Seven research assistants (senior registrars) were recruited and trained on perineal massage, hospital hands-on protocol, assessment of perineal injuries, standard labour ward care, and entering of inventory. They were subsequently rostered from Sunday to Saturday to attend to consenting eligible participants during the second stage of labour. The training sessions incorporated both theoretical and practical components, ensuring that each research assistant demonstrated proficiency in the technique before participating in the study. Additionally, periodic refresher sessions and supervisory assessments were conducted throughout the study period to maintain adherence to the standardised protocol.

### 2.8. Data and Statistical Analysis

Data was entered in an Excel sheet and cleaned. Continuous data was presented in mean and standard deviation and analysed using the *T*-test (for continuous parametric variables) or the Mann–Whitney *U*-test (for continuous nonparametric variables). The categorical variables were analysed using the chi-square test (or Fisher exact test) where appropriate. The Statistical Package for Social Sciences (SPSS) version 26 was used for data analysis. All analyses were done using the intention-to-treat approach. The statistical significance was inferred from a *p* value ≤ 0.05.

### 2.9. Study Procedure

Following informed written consent, the eligible participants were recruited. The participants were informed about the procedure in the antenatal clinic, informed consent obtained in the antenatal clinic, and re-confirmed on presentation at the labour and delivery ward of the hospital. They were admitted and randomised into either group. After randomization, the participants were anonymized. They were cared for and monitored in labour according to the study hospital's labour ward protocol.

In the second stage of labour, the women in the intervention group were placed in a dorsal position for the massage. The senior resident doctor therefore wore sterile gloves lubricated with 5 mL of sterile lubricant (*K*-*Y* Jelly) and massaged the perineum with a gentle up-down pressure towards the rectum in such a way that each movement lasted 1 min along the 3 o'clock and 9 o'clock positions (U-shaped reciprocating motion) in the second stage of labour. Overall, each participant in the intervention group was massaged for 10 min, and the degree of downward pressure was determined by the participants' responses, with the pressure being eased if they expressed pain or a burning sensation. Massage was stopped, and the affected participant dropped from the study if she wished to opt out. In both the intervention and control groups, the hospital hands-on protocol was applied, which includes guarding the perineum with a pad on the dominant hand and guiding and maintaining the flexion of the foetal head after crowning with the other hand. Mediolateral episiotomy was given at crowning in participants with an overstretched perineum that threatened to tear. The midwife on duty was used as the chaperone during the intervention, delivery, and outcome assessment. The control group did not receive any perineal massage during the second stage of labour.

**Figure 1:**
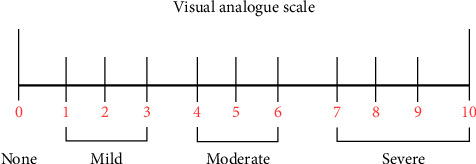
Visual analogue scale.

The two groups were assessed for perineal injuries after delivery. In addition, the labia, vaginal, and cervix were carefully inspected for tears. The rate of episiotomies in the groups was also assessed. The degree of spontaneous perineal tear, the duration of the second stage of labour (using a stop clock), and the birth weight of the babies using a weighing balance were all assessed in the two groups and recorded. Damage to the perineal skin and/or mucous membrane of the vagina was referred to as first-degree perineal laceration. Second-degree perineal laceration included those of perineal skin and/or mucous membrane of the vagina as well as involvement of perineal muscles; third-degree perineal laceration included perineal skin and/or mucous membrane of the vagina, perineal muscles as well as involvement of the anal sphincter; and fourth-degree perineal laceration included perineal skin and/or mucous membrane of the vagina, perineal muscles, anal sphincter as well as involvement of the rectal mucosa [17]. At 4 h and 24 h postpartum, the presence and severity of perineal pain were evaluated using a visual analogue scale, as shown in Figure 1. The participants were asked to tick the number that corresponds to the severity of their pain. Visual analogue scale (Figure 1), as developed by Portenoy and Tanner [20, 21], is graded from 0 to 10. One end of this simple pain evaluation instrument is a zero, and on the other end is 10, with zero representing no pain and 10 indicating severe pain. A patient may report one of four levels of pain: none (0), mild (1–3), moderate (4–6), or severe (7–10) by marking her pain perception on a scale of zero to 10 (see Figure 1).

### 2.10. Outcome Measures

The primary outcome measure was the proportion of participants having perineal tears during vaginal delivery between the intervention and control groups, while the secondary outcome measures were the proportion of participants having episiotomies during vaginal delivery, the mean duration of the second stage of labour, and perineal pain and its severity between the intervention and control groups. In this study, perineal outcomes refer to the status of the perineum after the baby is delivered (intact perineum, episiotomy with no or mild extension, episiotomy with marked extension, tears, degree of tears, and degree of perineal pain after the second stage of labour).

## 3. Results

**Figure 2:**
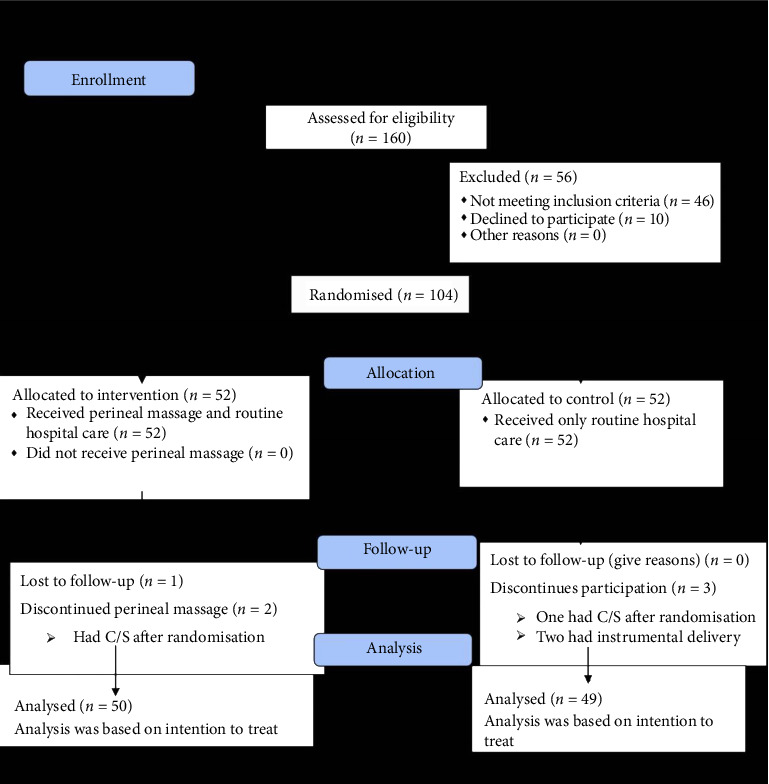
Consort/study flowchart.

The study was conducted from August 1, 2022, to January 31, 2023. A total of 160 participants were assessed for eligibility; 46 did not meet the inclusion criteria, and 10 declined to participate. One hundred and four participants were subsequently randomised into two groups: the intervention/perineal massage group (*n* = 52) and the control group (*n* = 52).  Figure 2 is the CONSORT flowchart showing the participants flow in the study. The socio-demographic characteristics of the participants in both arms of the study are shown in Table 1. There was no significant difference between the two groups regarding their baseline characteristics. Also there was no significant difference in the possible neonatal outcomes (impact factors) such as head circumference, birth weight, and first and fifth minute Apgar scores between the two groups (*p* > 0.05). This is shown in Table 1.

Table 2 shows the primary and secondary outcomes between the two groups. Overall perineal trauma in the intervention group was significantly lower than in the control group (27 (54.0%) vs. 40 (81.6%); RR = 0.66; 95% CI = 0.50–0.88; *p*=0.003). While there was no recorded case of peri-urethral laceration or third and fourth degree perineal tears in both groups, 8 (15.4%) and 6 (11.5%) of participants in the massage group had first-degree perineal tears and spontaneous second-degree perineal tears, respectively, when compared to 14 (26.1%) and 4 (7.7%) in the control group (*p* > 0.05). In addition, the episiotomy rate was not statistically significantly lower in the massage group compared to the control group (13 (26%) vs. 22 (44.9%), RR = 0.58; 95% CI = 0.33–1.02; *p*=0.06). Also, the mean duration of the second stage of labour in minutes was significantly lower in the massage group (83.1 ± 17.5 min) compared to the control group (94.2 ± 18.9 min; *p*=0.002).

In terms of comparison of the perception of pain at 4 h and 24 h, fewer participants in the massage group had moderate and severe perineal pain 4 h after delivery compared to the control group (10 (20%) vs. 25 (51%), *p*=0.001). In addition, the number of participants with 24 h postpartum moderate perineal pain was statistically significantly lower in the massage group than in the control group (5 (10%) vs. 14 (26.9%); *p*=0.019). This is shown in Table 3.

Table 4 shows the pain level (severity) evaluation between the two groups at 4 h and 24 h postpartum. The mean level of perineal pain at 4 h postpartum (4.3 ± 0.3 vs. 6.1 ± 0.5; *p*=0.03) and at 24 h postpartum (2.2 ± 0.1 vs. 4.2 ± 0.3; *p*=0.02) were significantly lower in the massage group than in the control group.

## 4. Discussion

The principal findings of this study were that perineal massage during the second stage of labour significantly reduces the proportion of overall perineal trauma, nonsignificantly decreases the rate of episiotomy, first or second degree perineal tears, significantly prevents and improves the severity of perineal pain, and shortens the duration of the second stage of labour but does not affect the first and fifth minute Apgar scores. The perineal trauma outcomes were also not affected by birth weight. Our findings are reassuring, as they show a significant beneficial effect of perineal massage on nulliparous parturients with singleton pregnancies undergoing vaginal births. None of the participants had third or fourth-degree perineal tears. Similar to our findings, a previous study by Karacam, Ekmen, and Çalişir [11] showed that there were no significant gains or drawbacks to perineal massage in terms of preventing individual perineal tears (first–second–third–fourth-degree tears). The perineal massage in their study was primarily done without a water-soluble lubricant to minimise possible frictions and abrasions. They also applied fundal pressure to aid delivery for some of the participants in their study. All these might have affected the outcome of their study. Also, a related previous study by Ibrahim, Elgzar, and Hassan [1] in Egypt demonstrated that, as compared to the standard care/control group, massaging the perineum with lubricant did not significantly lower the risk of spontaneous perineal tear or episiotomy during the second stage of labour. In line with our present study, a previous but similar study by Oglak and Obut [19] in Turkey found a significant reduction in the proportion of overall perineal trauma in participants who had perineal massage during the second stage of labour. However, it was noted in their study that first degree tears were higher in the massage group than in the control group, while second degree tears were lower in the massage group than in the control group. These were the direct opposite of the findings on first and second degree tears in this study. They started perineal massage at crowning, and that may affect the duration of the intervention and subsequently the outcomes. Lucena da Silva et al. and Aquino et al. in their systematic review concluded that perineal massage, are associated with a lower risk of perineal laceration [17, 18].

The nonsignificant reduction in episiotomy rates in the intervention group is contrary to the outcome of a previous study by Oglak and Lucena [19], which showed a significant reduction in episiotomy rates in the massage group compared to the control group. Also, contrary with our study, a Turkish study by Bayrakter and Başer [22] reported that perineal massage significantly decreases episiotomy rate (*p*=0.001). This was derived from their findings, which revealed that 34.3% of the participants in the massage group had episiotomies, while 48.6% of the participants in the control group had them. In another previous randomised controlled study by Romina et al. [9] that evaluated the effect of perineal massage on the episiotomy and laceration rates among nulliparous pregnant women, the massage group had a significantly decreased rate of episiotomy compared to the control group (*p* < 0.001). However, there was no statistically significant difference in perineal lacerations between the two groups. The nonsignificant difference observed in the Romina et al. study on perineal laceration rate, similar to ours, could be that the massage was started when the participants were in the active phase of labour and continued till delivery. In our data, perineal massage was applied to the parturient during the second stage of labour.

The present study showed a significant reduction in the incidence, severity, and duration of perineal pain in the perineal massage group compared to the control group. Most of the previous studies that showed a significant reduction in the proportion of perineal tears and episiotomy rate in the massage group compared to the control also documented a significant reduction in the incidence, severity, and length of perineal pain of the same trend [9, 10]. This outcome is probably because perineal massage enhances blood supply to the perineum, relaxes the perineal muscles, and softens and increases the elasticity of the tissues of the perineum [9]. The resultant effect is the prevention of perineal trauma and, by extension, perineal pain. Contrary to our findings, Karacam, Ekmen, and Çalişir [11] found that perineal massage had no effect on the incidence or severity of perineal pain postpartum. This contrary finding may not be unconnected to their methodology, in which perineal massage was done without lubricant and fundal pressure was frequently applied.

The average duration of the second stage of labour for nulliparous women is described in the literature between 60 and 120 min, and beyond the upper limit, the possibility of foetal distress and maternal exhaustion increases [23]. This present study showed a statistically significant reduction in the mean duration of the second stage of labour in the massage group compared to the control group (*p*=0.002). However, the reduction of the second stage duration in our study is still below the upper time limit. On the contrary, Karacam, Ekmen, and Çalişir [11] in a previous study recorded an overall shorter second stage duration but found no significant difference in the mean duration of the second stage of labour between the intervention and control groups (*p*=0.876). The outcome of their study might have been influenced by their use of fundal pressure. Also Dieb et al. [10], in a randomised controlled study to determine the use of perineal massage and pelvic floor muscle training for reducing perineal trauma in pregnant women older than 35 years, found a nonsignificant difference in the mean duration of the second stage of labour between the massage group and control groups (*p*=0.67). The result of the Dieb et al. study is at variance with the findings in our present study, probably because of the difference in inclusion criteria and methodology. Their participants were both nulliparous and multiparous women, and the intervention was that of self-administered antenatal massage and pelvic floor muscle training. In a meta-analysis of three randomised control trials, Aquino et al. [15] concluded that the use of perineal massage does not shorten the duration of the second stage of labour. In line with the findings of the present study, Oglak demonstrated a shorter duration of the second stage in the massage group compared to the control group.

The clinical implications of the study findings are that, depending on the severity of the trauma, perineal damage during vaginal birth may result in co-morbidities, including sexual dysfunction from dyspareunia and urine and faecal incontinence. Correspondingly, the outcomes of the current study may be most likely attributable to perineal massage, which relaxes the perineum and increases the suppleness of its muscles and tissues. This is because the application of this technique during the second stage of labour can transform the perineal tissues of nulliparous women from their originally rigid perineum to a more relaxed and elastic one, with a higher possibility of an intact perineum, a lesser incidence of perineal pain, and a reduction in the duration of labour. Lubrication with a water-soluble gel also ensures reduced friction during massage and hastens vaginal birth. What is not certain is whether perineal massage should be done only in the second stage of labour, throughout the active phase of labour, before the onset of labour, or both in the antepartum and intrapartum periods of the pregnancy [6, 9].

Another insight in the clinical implications for the study is the finding that second-degree lacerations were more frequent in the massage group, albeit without statistical significance, goes against the expected outcome if perineal massage were genuinely effective in reducing lacerations. Additionally, the juxtaposition of the percentage of episiotomies, which commonly lead to second-degree injuries, with the percentage of spontaneous second-degree lacerations is noteworthy. This second-degree injury additive phenomenon' reveals a striking similarity in second-degree perineal lacerations between the massage and control groups, with both hovering around 69% and 67%, respectively. This similarity in percentages might imply that the occurrence of second-degree perineal trauma, whether due to spontaneous lacerations or episiotomies, was essentially comparable between the groups, despite the intervention. However, one proviso was that perineal massage had a lower incidence of first-degree perineal tears in the study when compared to controls.

This study appears to demonstrate statistical significance but not clinical significance for the benefits of intrapartum perineal massage among nulliparous parturients. This is because the present study did not have any reported cases of third or fourth degree perineal tears, but reported only first degree and second degree lacerations as well as rates of episiotomies, which are often described as normal outcomes of vaginal delivery and heal very well when sutured correctly.

Proper care of the perineum during childbirth is crucial to prevent injuries. In addition to perineal massage, other interventions have been employed, including warm or cold compresses, and perineal management techniques [24]. While single techniques have shown mixed results, emphasis are now placed on combining multiple methods, known as “intrapartum bundles and interventions,” aimed at reducing severe perineal trauma and increasing the rate of intact perineum. These bundles have proven more effective in minimizing perineal injuries during vaginal deliveries [25].

The strength of our study is that it was conducted among nulliparous women, ensuring homogeneity, and that previous vaginal delivery as a confounder was eliminated. In addition, the study is a randomised controlled trial; hence, allocation and selection bias were eliminated via randomization and group concealment. Although this study provides important insights, some limitations were noted. The short period of follow-up in our study might have limited further assessment of perineal pain beyond 24 h postpartum. Also, the study participants were not blinded because of the nature of the intervention, so a placebo effect on patient-reported pain scores is possible. The results on the correlation between perineal massage and perineal tears may be lowered by the high occurrence of episiotomies. While our study addresses perineal massage, other factors contributing to perineal trauma should be explored in future studies. The study was single-centre-based; hence, a large multi-centre randomised controlled trial is advocated to validate our findings. We were unable to perform both univariate and multiple logistic regression analyses because this study is a randomized controlled trial, and the baseline socio-demographic characteristics were homogeneous across the study groups.

## 5. Conclusion

Intrapartum perineal massage has a beneficial effect on nulliparous parturients with singleton pregnancy undergoing spontaneous vaginal births. It significantly decreased the risk of overall perineal trauma, perineal pain, and duration of the second stage of labour. Nulliparous women should be counselled on the potential benefits of intrapartum perineal massage, and obstetricians are encouraged to provide the technique to consenting women.

## Figures and Tables

**Table 1:** Socio-demographics characteristics of all the participants and neonatal outcomes between the perineal massage and control group.

Variables	Perineal massage (*n* = 52)	Control (*n* = 52)	*X* ^2^	*p* value
Marital status				
Married	49 (94.2)	47 (90.4)	0.542	0.46
Single	3 (5.8)	5 (9.6)		
Widowed	0	0		
Divorced	0	0		
Educational level				
Primary	7 (13.5)	7 (13.5)	1.323	0.52
Secondary	34 (65.4)	29 (55.8)		
Tertiary	11 (21.2)	16 (30.8)		
Resident location				
Rural	44 (84.6)	39 (75)	1.492	0.22
Urban	8 (15.4)	13 (25)		
Occupation				
Artisan	3 (5.8)	5 (9.6)	4.957	0.29
Civil servant	24 (46.2)	17 (32.7)		
Housewife	13 (25)	12 (23.1)		
Student	2 (3.8)	10 (19.2)		
Trader	10 (19.2)	8 (15.4)		

**Maternal characteristics**			***T* value**	**p** **value**

BMI	28.6 ± 4.3	29.5 ± 5.3	−0.565	0.89
Gestational age in weeks	38 ± 1.3	37.8 ± 1.2	1.191	0.24

**Neonatal outcome/impact factor**			**X** ^2^ **/*T***	**p** **value**

Birth weight	3.4 ± 0.2	3.4 ± 0.3	0.14	0.89
Head circumference	34.5 ± 1.5	34.1 ± 1.7	1.34	0.18
1^st^ minute apgar score				
6/10	9 (18)	12 (24.5)	0.82	0.66
7/10	19 (38)	19 (38.8)		
8/10	22 (44)	18 (36.7)		
5^th^ minute apgar score				
8/10	9 (18)	12 (24.5)	0.62	0.43
9/10	41 (82)	37 (75.5)		

*Note:* “Values in *n* (%).”

Abbreviation: BMI = body mass index.

**Table 2:** Primary and secondary outcomes between the perineal massage group and control group.

Outcome measures	Perineal massage (*n* = 50)	Control (*n* = 49)	*X* ^2^/*T* (RR; 95% CI)	*p* value
Overall perineal trauma	27 (54)	40 (81.6)	8.638 (0.66; 0.50–0.88)	0.003
Intact perineum	23 (46)	9 (18.4)		
Spontaneous tears				
1^st^ degree	8 (57.1)	14 (77.8)	6.206 (0.56; 0.26–1.21)	0.14
2^nd^ degree	6 (42.9)	4 (22.2)	0.742 (1.23; 0.37–4.10)	0.33
3^rd^ degree	0	0		
4^th^ degree	0	0		
Total	14 (100)	18 (100)		
Episiotomy				
Yes	13 (26)	22 (44.9)	3.867 (0.58; 0.33–1.02)	0.06
No	37 (74)	27 (55.1)		
Rate of spontaneous tears in the whole group	14/50 (28)	18/49 (36.7)		
Rate of spontaneous tears in parturients with perineal trauma	14/27 (51.9)	18/40 (45)		
Perineal pain 4 h postpartum				
Yes	19 (38)	36 (73.5)	12.609 (0.52; 0.35–0.77)	< 0.001
Perineal pain 24 h postpartum				
Yes	8 (16.0)	21 (42.9)	8.618 (0.37; 0.18–0.76)	0.003
Mean duration of 2^nd^ stage of labour (minutes)	83.1 ± 17.5	94.2 ± 18.9	−3.116	0.002

*Note:* “Values in *n* (%).”

**Table 3:** Comparison of the perception/degree of pain between perineal massage group and control group at 4 h and 24 h.

Perineal pain assessed with VAS	Perineal massage (*n* = 50)	Control (*n* = 49)	*X* ^2^	*p* value
At 4 h postpartum				
Mild pain (1–3)	40 (80)	24 (49)	13.77	0.001
Moderate pain (4–6)	6 (12)	6 (12.2)		
Severe pain (7–10)	4 (8)	19 (38.8)		
At 24 h postpartum				
Mild pain (1–3)	45 (90)	35 (73.1)	5.50	0.002
Moderate pain (4–6)	5 (10)	14 (26.9)		
Severe pain (7–10)	0 (0)	0 (0)		

*Note:* “Values in *n* (%).”

Abbreviation: VAS = visual analogue score.

**Table 4:** Pain evaluation between the two groups at 4 h and 24 h postpartum.

Timing of pain evaluation	Perineal massage	Control	*T* value	*p* value
Mean level of pain at 4 h	4.3 ± 0.3	6.1 ± 0.5	−2.718	0.03
Mean level of pain at 24 h	2.2 ± 0.1	4.2 ± 0.3	−3.121	0.02

## Data Availability

Data available on request from authors.
